# Transcriptome-phenotype matching analysis of how nitrogen sources influence *Lacticaseibacillus rhamnosus* tolerance to heat stress and oxidative stress

**DOI:** 10.1186/s12934-022-01985-0

**Published:** 2022-12-12

**Authors:** Chenchen Zhang, Haohao Cheng, Yuemei Han, Yunchao Wa, Dawei Chen, Chengran Guan, Yujun Huang, Ruixia Gu

**Affiliations:** 1 College of Food Science and Engineering, Yangzhou University, 196 Huayang Xilu, Yangzhou, 225100 People’s Republic of China; 2 Jiangsu Key Laboratory of Dairy Biotechnology and Safety Control, Yangzhou, People’s Republic of China; 3 Jiangsu Dairy Biotechnology Engineering Research Center, Yangzhou, People’s Republic of China

**Keywords:** *Lacticaseibacillus rhamnosus*, Heat stress, Oxidative stress, Nitrogen source, Carbon catabolite repression

## Abstract

**Background:**

Spray drying is the most cost-effective production method for lactic acid bacteria starters, but heat and oxidative stresses result in low survival rates. The heat stress and oxidative stress tolerance of *Lacticaseibacillus rhamnosus* cultured in tryptone-free MRS (NP-MRS) broth was much stronger than that in MRS or tryptone-free MRS broth supplemented with phenylalanine (Phe-MRS). Here, multiple transcriptome-phenotype matching was performed on cells cultured in NP-MRS, MRS and Phe-MRS broths to reveal the mechanism by which nitrogen sources influence *L. rhamnosus* tolerance to heat stress and oxidative stress.

**Results:**

Compared with cells cultured in NP-MRS broth, 83 overlapping differentially expressed genes (DEGs) were downregulated by either tryptone or phenylalanine. The overlapping DEGs were mainly classified into carbohydrate metabolism and membrane transport pathways, which are often repressed by glucose during carbon catabolite repression (CCR). In the presence of glucose, the heat stress or oxidative stress tolerance of *L. rhamnosus* hsryfm 1301 was not strengthened by supplementation with secondary carbohydrates. Replacing glucose with mannose, fructose or ribose improved the heat stress and oxidative stress tolerance of *L. rhamnosus* hsryfm 1301 (5 to 46-fold).

**Conclusions:**

Alleviation of CCR might be a reason for the resistance of *L. rhamnosus* hsryfm 1301 to heat stress and oxidative stress in a low-nitrogen environment. The survival rate of *L. rhamnosus* during spray drying will hopefully be improved by relieving CCR. It is a new discovery that nitrogen sources influence CCR in *L. rhamnosus*.

## Background

Probiotics provide a number of health benefits. Viable count is essential for lactic acid bacteria (LAB) to achieve fermentation and beneficial effects. However, various stresses that LAB encounter during the production, transport and storage processes can seriously decrease their viable count. Several attempts, such as freezing, drying and microencapsulation, have been made to improve the viability of probiotics in different food products during their production until the time of consumption [1].

An organism survives stress conditions through multiple physiological responses. Comparative genomics, DNA microarrays, transcriptome and proteome profiling, and genotype–phenotype matching have been employed to identify genetic factors regulated during single stress exposure [2]. Regulators such as HrcA, CtsR, GlnR, and CcpA, and the classical heat shock proteins GroEL and DnaK, play an important role in the stress resistance of LAB [3–5].

However, there are disadvantages in the study of single stress tolerance. LAB usually encounter various stress conditions during production and storage processes, but the study of single stress tolerance can only provide information for treating one form of stress. On the other hand, hundreds of functional genes and regulatory elements respond to one stress in LAB [6, 7], so it is difficult to screen novel major genes by single stressome-phenotype matching. Therefore, cross-adaptation which results from stress response overlaps occurring among different stress treatments, has been applied to improve resistance and reveal the underlying mechanisms of stress responses [8–10].

Spray drying is cost-effective and highly flexible and has been applied in the production of LAB powder [11, 12]. Heat stress and oxidative stress are the major stresses resulting in a low survival rate of starter cultures during spray drying [13, 14]. In our previous study, mutual cross-adaptation of *Lacticaseibacillus rhamnosus* hsryfm 1301 to oxidative stress and heat stress was found [15]. Transcriptome-phenotype matching based on RNA sequencing was implemented, and it was revealed that the high proportion of transcriptional homogenization, especially the decrease in abundance of nitrogen source transporter and metabolism enzyme genes was a reason for the cross-adaptation [10]. Tryptone-free MRS broth universally enhanced the heat stress and oxidative stress tolerance (H-OST) of *L. rhamnosus* cells, with the survival rates under heat stress and oxidative stress being increased by 130 and 40 times, respectively (OD_600_, 2.0). The spray-drying survival rate of *L. rhamnosus* hsryfm 1301 cultured in tryptone-free MRS broth rose to 75% (from 30%), and the spray-dried powder also exhibited higher survival in simulated gastrointestinal digestion. After supplementing tryptone-free MRS broth with phenylalanine, isoleucine, glutamate, valine, histidine, or tryptophan, the H-OST of *L. rhamnosus* hsryfm 1301 exhibited a sharp drop [16]. Amino acids often played a positive role in stress resistance of LAB [9, 17]. Therefore, it is of interesting to determine how the decrease in abundance of nitrogen sources improves the survival of *L. rhamnosus* hsryfm 1301 under heat stress and oxidative stress.

In the present study, *L. rhamnosus* hsryfm 1301 was cultured in MRS, tryptone-free MRS and tryptone-free MRS supplemented with phenylalanine to enable transcriptome-phenotype matching and screen gene transcription profiles which were correlated to survival characteristics, revealing the mechanism of the cotolerance of *L. rhamnosus* to heat stress and oxidative stress through the downregulation of nitrogen metabolism.

## Results

### Correlation of expression patterns in MRS, NP-MRS and Phe-MRS broths

**Fig. 1:**
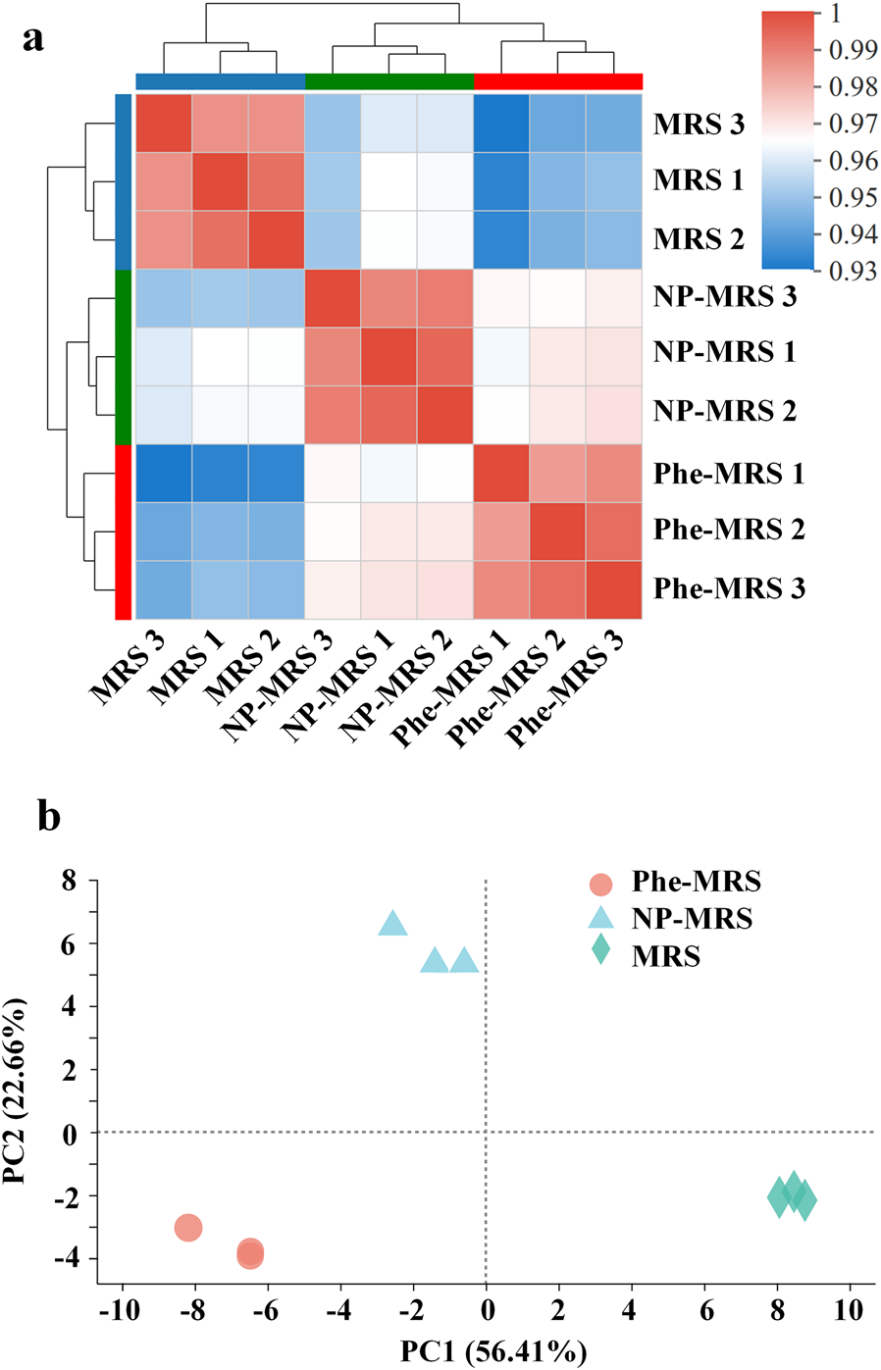
Correlation of expression patterns in different media. **a** Pairwise correlation of different biological replicates from the MRS, NP-MRS and Phe-MRS groups. The color intensities (scale in the side bar) and the numbers indicate the degree of pairwise correlation. **b** PCA of RNA-seq

The reads obtained from sequencing were mapped to the genome of *L. rhamnosus* hsryfm 1301. The pairwise correlation between each biological replicate was shown based on normalized expression levels. A high reproducibility among biological replicates from the same medium was observed, indicated by a Pearson correlation > 0.98. Moreover, when comparing the results among different media, a Pearson correlation < 0.97 can be obtained (Fig. 1a). Both the Pearson correlation and the PCA (Fig. 1b) of RNA-sequencing indicated the high reproducibility among technical replicates of RNA-seq and the differences in transcription profiles induced by tryptone and Phe. Interestingly, although the H-OST of cells from the Phe-MRS were more similar to those from the MRS, the transcription profile correlation between the Phe-MRS group and the NP-MRS group was higher than that between the Phe-MRS group and the MRS group (Fig. 1).

### Transcriptional regulation overlaps in response to tryptone and Phe

**Fig. 2:**
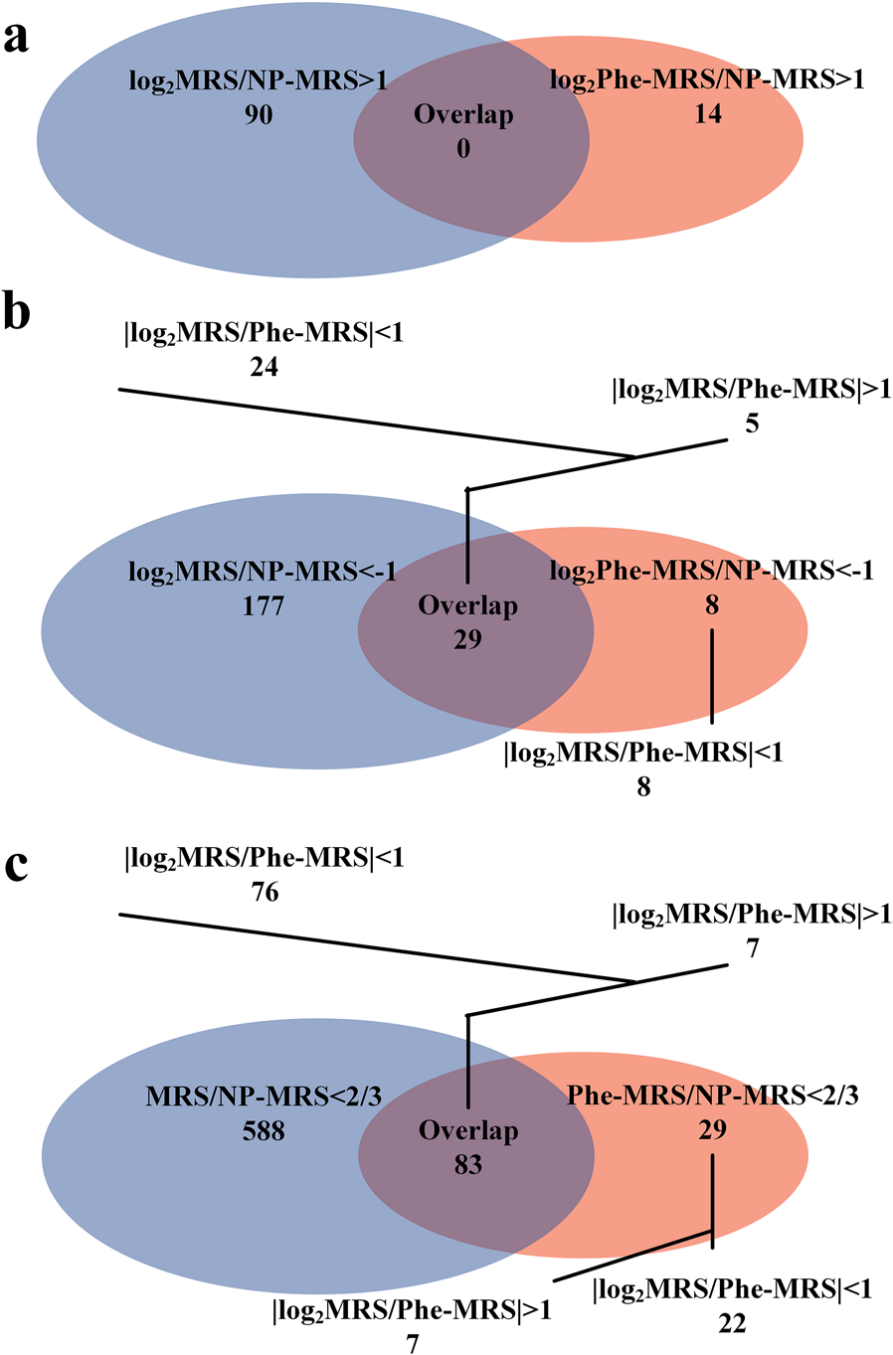
Overlapping DEGs of *L. rhamnosus* hsryfm 1301 between the MRS group vs. NP-MRS group comparison and the Phe-MRS group vs. NP-MRS group comparison. **a** |log_2_FC|> 1, upregulation. **b** |log_2_FC|> 1, downregulation. **c** FC < 2/3, downregulation

In our previous study, it was found that the H-OST of *L. rhamnosus* hsryfm 1301 cells cultured in MRS broth and Phe-MRS broth was significantly lower than those in NP-MRS broth. Overlapping transcriptional regulation in response to tryptone and Phe would be helpful to understand this phenomenon. To facilitate data presentation, the NP-MRS group was described as a control. Compared with the NP-MRS group, 90 genes were upregulated (log_2_FC > 1) in the MRS group, and 14 genes were upregulated (log_2_FC > 1) in the Phe-MRS group (Fig. 2a). There was no upregulation overlap between the MRS vs. NP-MRS comparison and the Phe-MRS vs. NP-MRS comparison. There were 206 genes downregulated (log_2_FC < -1) in the MRS group. Thirty-seven genes were downregulated (log_2_FC < -1) by Phe, among which 29 genes were overlapping DEGs (log_2_FC < -1) responding to both tryptone and Phe (Fig. 2b), and the remaining 8 genes were also slightly downregulated (log_2_(2/3) < log_2_FC < -1) by tryptone.

To find more overlapping DEGs, the downregulated genes were also analyzed under the standard of |log_2_FC|> 0.585 (FC < 2/3). There were 671 genes downregulated (log_2_FC < -0.585) in cells cultured in MRS broth and 112 genes were downregulated (log_2_FC < -0.585) by Phe, among which 83 genes were overlapping DEGs (log_2_FC < -0.585) responding to both tryptone and Phe (Fig. 2c). It was suggested that the lower H-OST of *L. rhamnosus* hsryfm 1301 in the MRS and Phe-MRS groups resulted from the genes downregulated by tryptone and Phe.

### KEGG analysis of overlapping DEGs in response to tryptone and Phe

**Fig. 3:**
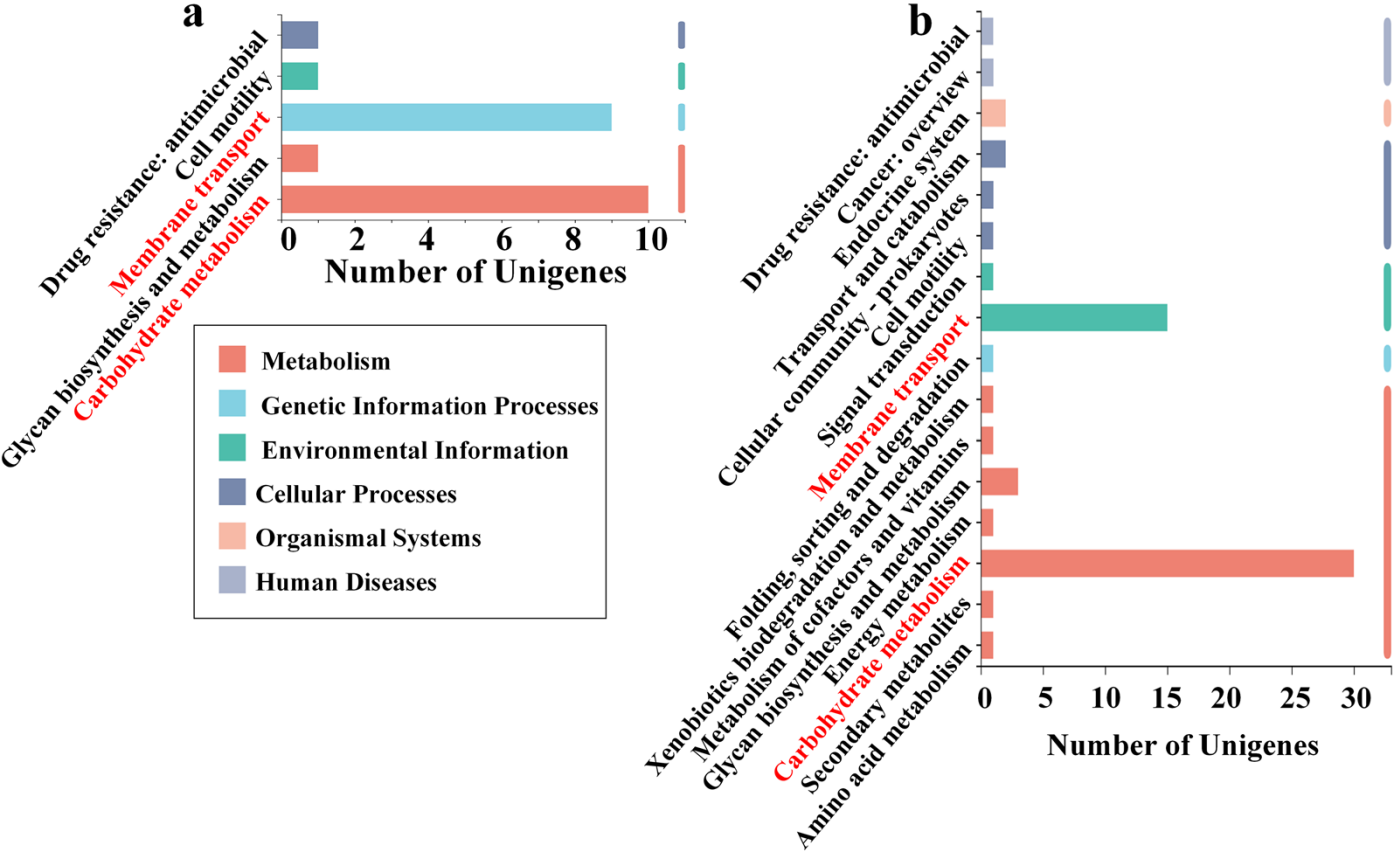
KEGG pathway analysis of the overlapping DEGs. **a** |log_2_FC|> 1. **b** FC < 2/3 (|log_2_FC|> 0.585)

Under the standard of |log_2_FC|> 1, 22 overlapping DEGs were involved in carbohydrate metabolism (10 DEGs), membrane transport (ABC transporters, 9 DEGs), drug resistance (1 DEG), cell motility (1 DEG), and glycan biosynthesis and metabolism (1 DEG) (Fig. 3a). All 9 overlapping membrane transport DEGs were related to carbohydrate transport. The 10 overlapping carbohydrate metabolism DEGs and 9 overlapping membrane transport DEGs were distributed in the metabolism pathways of 6 common sugars.

Under the standard of |log_2_FC|> 0.585, 83 overlapping DEGs were enriched 63 times (the functions of some DEGs were unknown and some DEGs were enriched in more than 1 pathway) and involved in 16 KEGG pathways (Fig. 3b). The enriched overlapping DEGs were mainly involved in carbohydrate metabolism (30 DEGs), membrane transport (ABC transporters, 15 DEGs), glycan biosynthesis and metabolism (3 DEGs), transport and catabolism (2 DEGs), and the endocrine system (2 DEGs) (Fig. 3b). All 15 overlapping membrane transport DEGs were presumed to encode proteins transporting carbohydrates according to NCBI NR annotation. The 30 overlapping carbohydrate metabolism DEGs and 15 overlapping membrane transport DEGs were distributed in the metabolism pathways of more than 10 common carbohydrates.

### Overlapping DEGs related to classical stress tolerance

**Fig. 4:**
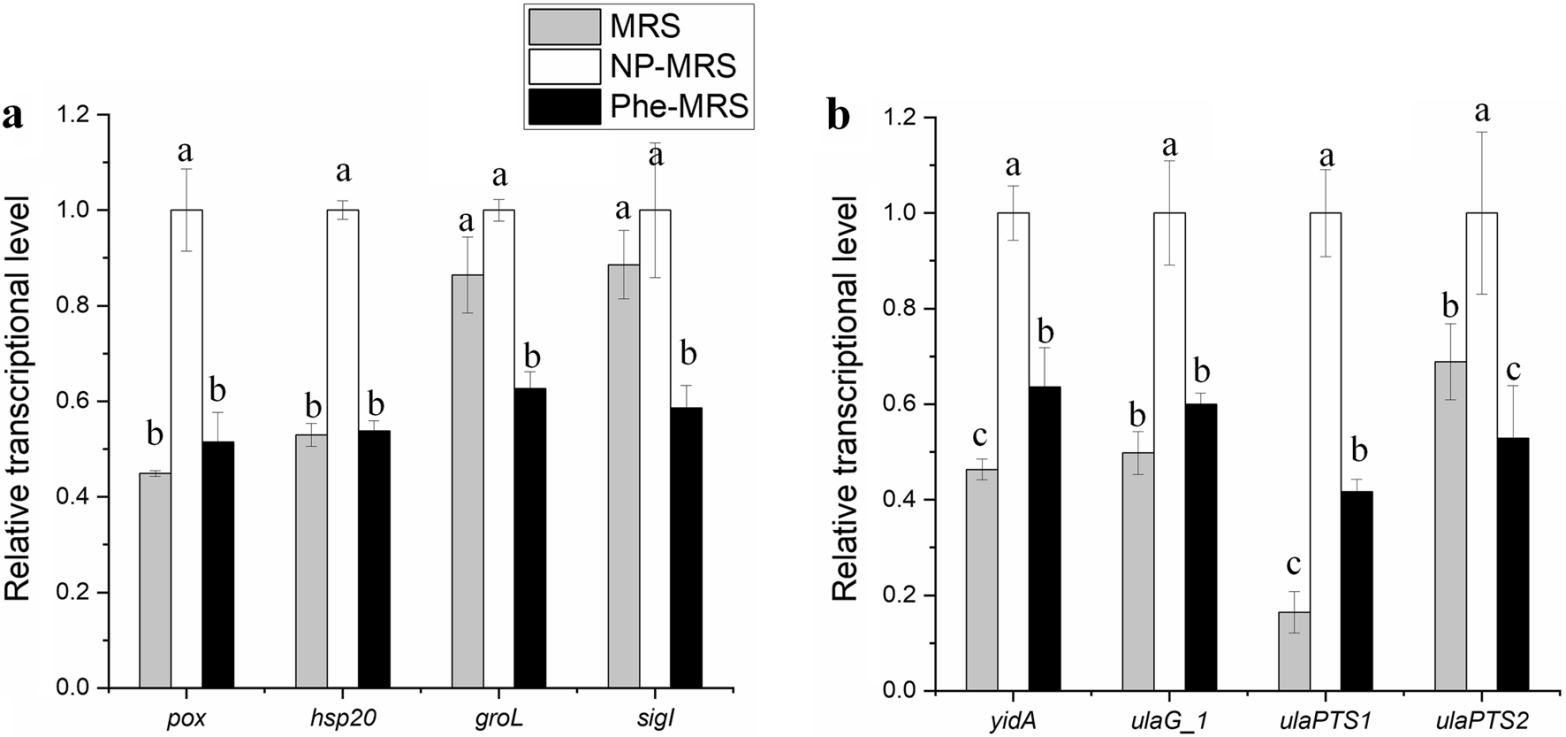
Overlapping DEGs related to classical stress tolerance. **a** Classical stress tolerance genes. **b** Genes in the ascorbic acid operon. ^a−^.^c^Values within each column with different superscripts are significantly different (*P* < 0.05)

Although the H-OST of cells from the Phe-MRS and MRS groups were far weaker than those from the NP-MRS group, only a few overlapping DEGs were found to be classical stress tolerance genes. One heat shock response (HSR) gene, *hsp20* was downregulated significantly by both tryptone and Phe, while *groL* and *sigI/σ*^*70*^ were downregulated by only Phe (Fig. 4a). Several genes considered to be relevant to the oxidative shock response (OSR), such as *pox/spxB* and the ascorbic acid operon, were downregulated by both tryptone and Phe. The transcription levels of most classical stress tolerance genes, such as regulators *(ctsR, hrcA,* and *ccpA*), chaperonins (*dnaK* and *dnaJ*), DNA repair genes (*recA, uvrB,* and *uvrC*) and proteases (*clpP*, *clpB*, and *ftsH*), were not influenced by tryptone or Phe. The regulation of classical HSR and OSR genes was unable to explain the cotolerance of *L. rhamnosus* to heat stress and oxidative stress in a low nitrogen source environment.

### Overlapping DEGs related to sugar utilization and pyruvate flow

**Fig. 5:**
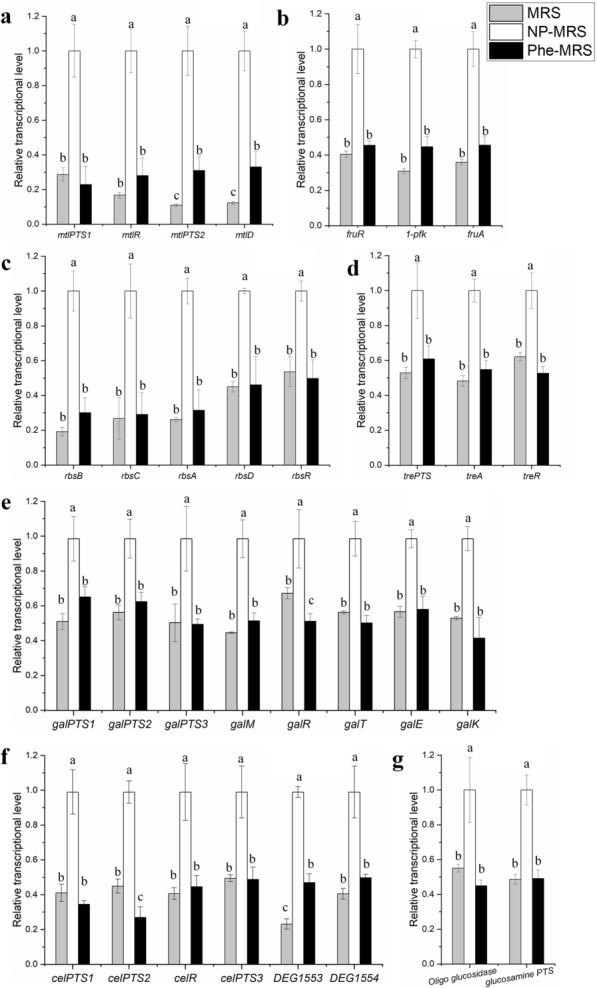
Overlapping DEGs related to secondary carbohydrates. **a** Genes in the mannose operon. **b** Genes in the fructose operon. **c** Genes in the ribose operon. **d** Genes in the trehalose operon. **e**. Genes in the galactose operon. **f** Genes in the cellobiose operon. **g** Genes in the isomaltose operon. ^a−^.^c^Values within each column with different superscripts are significantly different (*P* < 0.05)

**Fig. 6:**
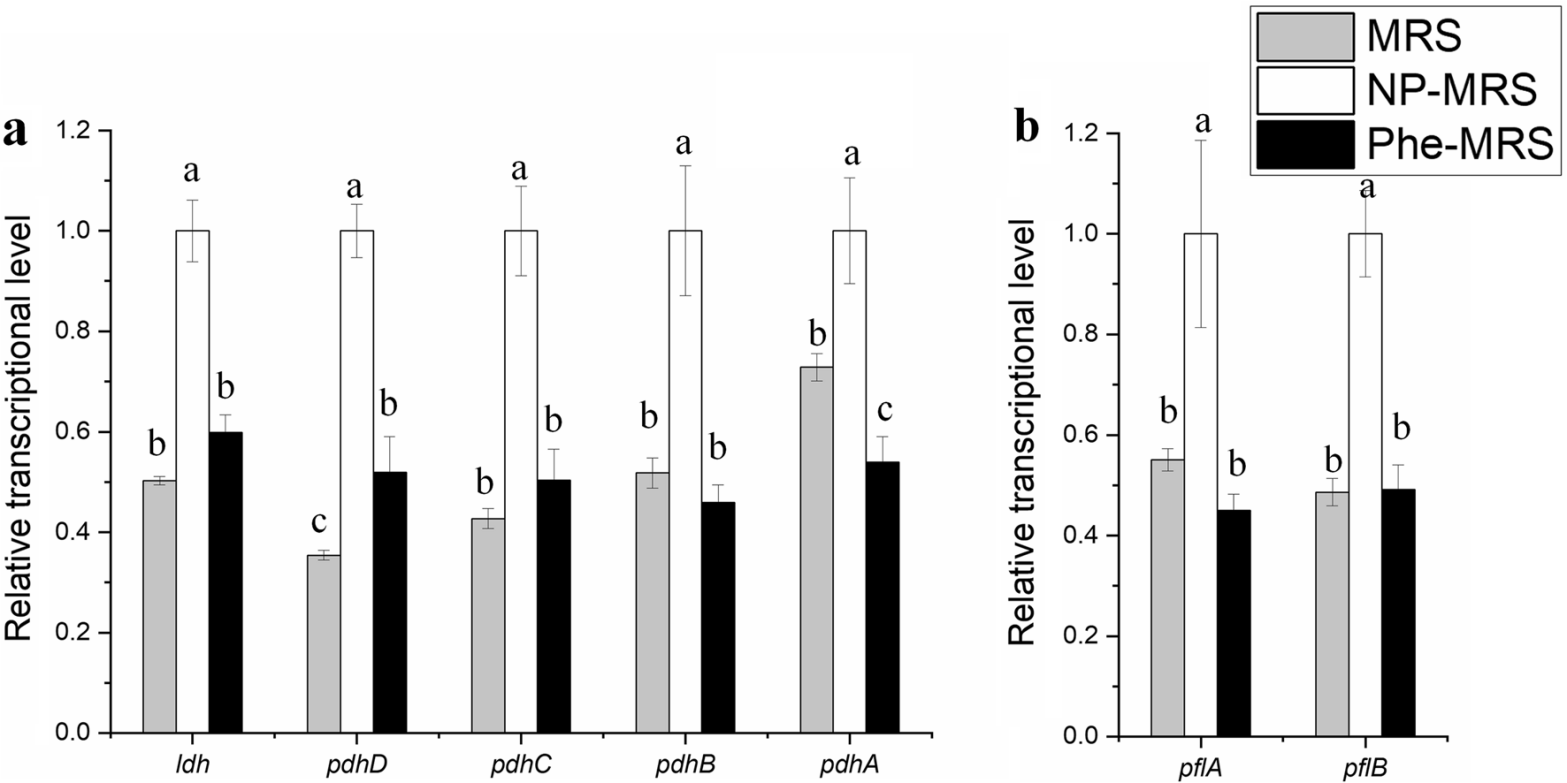
Overlapping DEGs related to pyruvate flow. **a** Genes related to acetate fermentation. **b** Genes related to formate fermentation. ^a−^.^c^Values within each column with different superscripts are significantly different (*P* < 0.05)

Most of the overlapping DEGs were related to the utilization of carbohydrates. Specifically, 4 genes in the *mlt* operon (mannose metabolism) were upregulated by more than threefold in a low nitrogen environment (Fig. 5a). Operons related to the utilization of fructose (Fig. 5b), ribose (Fig. 5c), trehalose (Fig. 5d), galactose (Fig. 5e), cellobiose (Fig. 5f) and isomaltose (Fig. 5g) were downregulated by both tryptone and Phe. These results suggested that *L. rhamnosus* hsryfm 1301 consumes a diversity of carbon sources under low nitrogen conditions. On the other hand, genes related to mixed acid fermentation, such as the *pdh* operon (acetate fermentation, Fig. 6a) and *pfl* operon (formate fermentation, Fig. 6a), were also downregulated by both tryptone and Phe. Moreover, most of these genes were similarly expressed in the MRS and NP-MRS groups. Both multiple carbon sources consumption and mixed acid fermentation were indicators of carbon catabolite repression (CCR).

### Heat stress and oxidative stress tolerance of *L. rhamnosus* hsryfm 1301 under conditions with different sugars

**Fig. 7:**
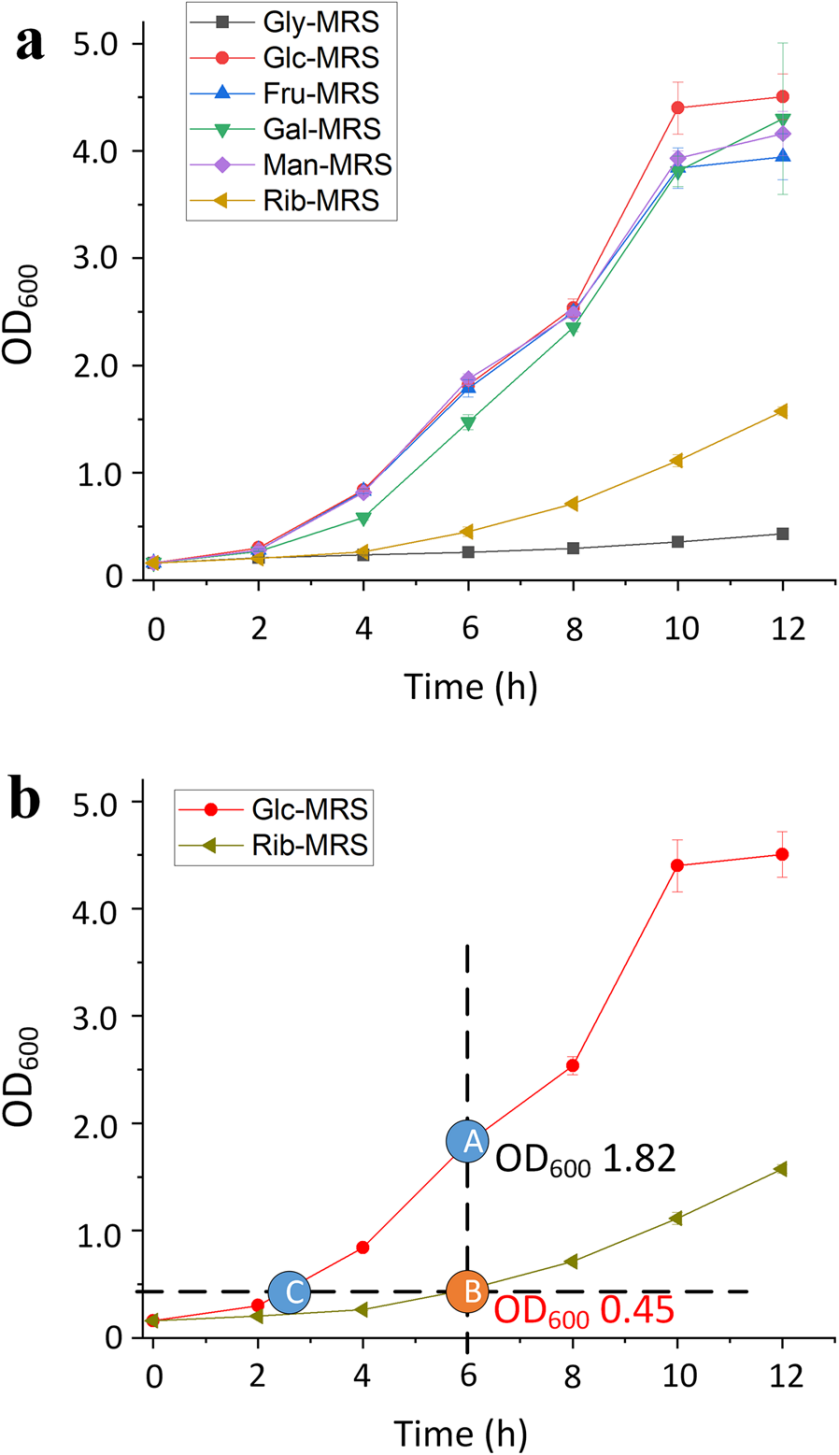
Growth characteristics of *L. rhamnosus* hsryfm 1301 under different carbon source conditions. **a** Growth curves. **b** Measurement points A, B and C were determined according to the Materials and methods section; Measurement points B and C, OD_600_, 0.45

Several genes related to multiple carbon source consumption and mixed acid fermentation were upregulated in a low nitrogen environment. These genes were often regulated by glucose during CCR. To identify whether this response involved intake of secondary carbon sources or was just performance of the carbon catabolite derepression, the H-OST of *L. rhamnosus* hsryfm 1301 with different carbon sources were investigated. In Man-MRS, Fru-MRS, and Gal-MRS broths, *L. rhamnosus* hsryfm 1301 could grow as well as in Glc-MRS broth, in which the OD_600_ value reached 1.8 in 6 h (Fig. 7a). However, *L. rhamnosus* hsryfm 1301 grew much slower in Rib-MRS broth and its OD_600_ reached 0.45 at 6 h, so the survival rate of *L. rhamnosus* hsryfm 1301 in Glc-MRS broth at OD_600_ 0.45 was also measured (Fig. 7b).

**Fig. 8:**
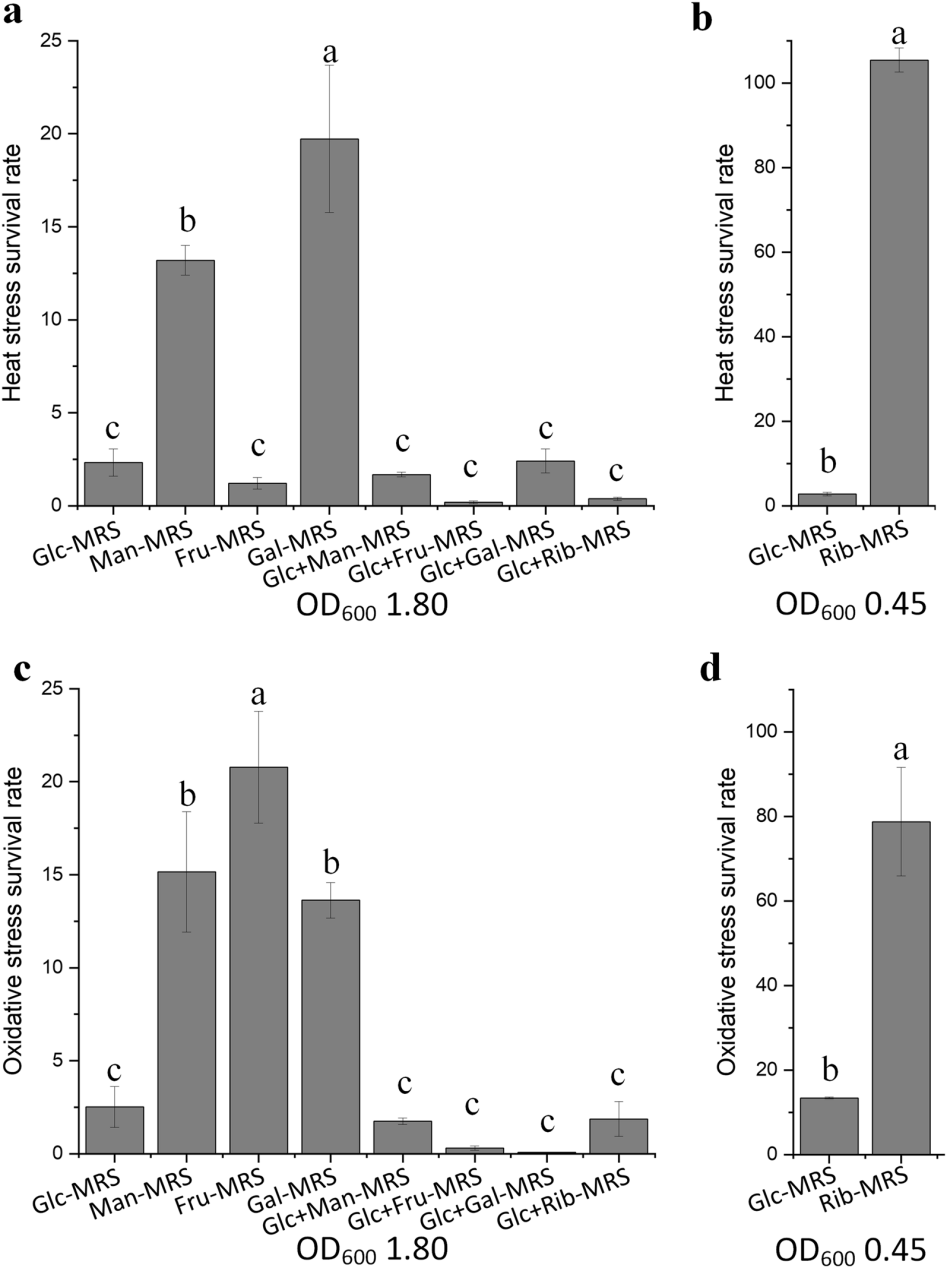
Heat stress and oxidative stress tolerance of *L. rhamnosus* hsryfm 1301 under different carbon source conditions. **a** Heat stress tolerance at OD_600_ 1.80. **b** Heat stress tolerance at OD_600_ 0.45. **c** Oxidative stress tolerance at OD_600_ 1.80. **d** Oxidative stress tolerance at OD_600_ 0.45. ^a−^.^c^Values within each column with different superscripts are significantly different (*P* < 0.05)

In the case of heat shock, the survival rate of *L. rhamnosus* hsryfm 1301 (OD_600_ 1.80) was 2.32% in Glc-MRS broth. After supplementation with mannose, fructose, galactose, or ribose (in Glc + Man-MRS, Glc + Fru-MRS, Glc + Gal-MRS, or Glc + Rib-MRS broths), the heat stress tolerance of *L. rhamnosus* hsryfm 1301 was not enhanced. However, when the glucose of Glc-MRS was replaced by mannose, galactose, or ribose (changing the broth to Man-MRS broth, Gal-MRS broth, or Rib-MRS broth, respectively), the thermotolerance of *L. rhamnosus* hsryfm 1301 was significantly enhanced (Fig. 8a). Specifically, the survival rate of *L. rhamnosus* hsryfm 1301 in Rib-MRS broth (OD_600_ 0.45) reached 105% (Fig. 8b). In the case of oxidative shock, treatment with H_2_O_2_ killed 97.21% of *L. rhamnosus* hsryfm 1301 cells in Glc-MRS broth (OD_600_ 1.80). The survival rate of *L. rhamnosus* hsryfm 1301 in Man-MRS, Fru-MRS, and Gal-MRS broths rose to 15.15%, 20.78%, and 13.63%, respectively (Fig. 8c). The survival rates of *L. rhamnosus* hsryfm 1301 at OD_600_ 0.45 in Glc-MRS and Rib-MRS broths were 13.40% and 78.74%, respectively (Fig. 8d). However, aerotolerance was not improved in Glc + Man-MRS broth, Glc + Fru-MRS broth, Glc + Gal-MRS broth, or Glc + Rib-MRS broth, which were the broths supplemented with different sugars (Fig. 8c). These results suggested that sugar replacement, not sugar supplementation improved the H-OST of *L. rhamnosus* hsryfm 1301.

## Discussion

*L. rhamnosus* is one of the most commonly used probiotics [18–20]. To keep *L. rhamnosus* cells alive to perform health promotion functions, many studies have been carried out to improve its stress tolerance. Bacteria can withstand harsh conditions and sudden environmental changes through stress-sensing systems [10]. Omics data-phenotype matching approaches are practical methods for identifying novel genes that respond to stresses in *L. rhamnosus* [10, 21–23]. However, the stress tolerance of LAB is achieved by a complex regulatory network containing hundreds of functional genes and regulatory elements [6, 7], so it is difficult to screen novel genes by single stressome-phenotype matching. Compared with single stress tolerance studies, the study of the response of LAB to complex stresses was more powerful to solve the loss of activity caused by multiple stress conditions.

In our previous study, it was found that *L. rhamnosus* hsryfm 1301 was able to cotolerate heat stress and oxidative stress by decreasing the abundance of nitrogen sources [10]. The H-OST of *L. rhamnosus* hsryfm 1301 cultured in NP-MRS broth was significantly stronger than that from MRS broth or NP-MRS broth supplemented with phenylalanine, isoleucine, glutamate, valine, histidine, or tryptophan. The spray drying survival rate of *L. rhamnosus* hsryfm 1301 cultured in NP-MRS broth rose to 75% (from 30% in MRS broth) [16]. Therefore, these results helped reveal the underlying mechanisms of stress responses. In the present study, the mechanism of the cotolerance of *L. rhamnosus* to heat and oxidative stresses through the downregulation of nitrogen metabolism was investigated.

In the MRS and Phe-MRS groups, *L. rhamnosus* hsryfm 1301 performed similarly and had much weaker H-OST than that in the NP-MRS group. After culturing *L. rhamnosus* hsryfm 1301 in MRS broth, Phe-MRS broth, or NP-MRS broth, the stressomes in the MRS and Phe-MRS groups were compared with the stressomes in the NP-MRS group, and then the regulation overlaps facilitated the screening of novel gene functions in response to stress.

Compared with the NP-MRS group, the number of regulated genes (|log_2_FC|> 1) in the MRS group was much greater than that in the Phe-MRS group, which was consistent with the composition complexity of the media. There was no upregulation overlap between the MRS vs. NP-MRS comparison and the Phe-MRS vs. NP-MRS comparison. Among the 206 genes downregulated in the MRS group and 37 genes downregulated by Phe, 29 genes were overlapping DEGs. It was suggested that the weaker H-OST of *L. rhamnosus* hsryfm 1301 in the presence of tryptone and Phe were due to gene down-regulation, and less attention should be given to the upregulated DEGs. Through multiple transcriptome-phenotype matching, the scope in gene selection was decreased. If single transcriptome-phenotype matching is implemented, a large number of genes will be found. For example, 746 genes of *Limosilactobacillus fermentum* were regulated under bile stress [6], and 506 differently expressed proteins were identified in *Lactiplantibacillus plantarum* under cold stress [7]. Because the FC of many DEGs was close to 2.0, the standard that |log_2_FC|> 1 might result in omissions of underlying DEGs. So, the downregulated genes were also analyzed under the standard FC < 2/3. As a result, 83 genes were overlapping DEGs responding to both tryptone and Phe.

Although amino acids can influence the stress resistance of LAB [9, 17], almost no overlapping DEGs (FC < 2/3) belonged to the amino acid metabolism pathway, and only few overlapping DEGs were related to classical stress tolerance. Some common stress response genes, such as regulators *(ctsR, hrcA,* and *ccpA*), chaperonins (*dnaK*, *dnaJ*, *groES*, and *groEL*) and proteases (*clp* and *ftsH*), respond to heat stress in LAB [2]. A theoretical analysis showed that HSR repressors (HrcA and CtsR), alternative *σ* factors, protein repair systems (chaperonins and proteases) and proteins involved in DNA repair and export of toxic substances were related to HSR in *Lacticaseibacillus casei* and *L. rhamnosus* [4]. On the other hand, genes related to detoxification of ROS (*sod*, peroxidase gene, *noxE*, and so on) and DNA repair (*recA*, *uvrA*, and *uvrB*) are often found in the OSR of LAB [2]. However, only one HSR gene, *hsp20*, was downregulated significantly by both tryptone and Phe, and several genes considered to be relevant to OSR, such as *pox/spxB* [24] and the ascorbic acid operon, were downregulated by both tryptone and Phe. The classical stress tolerance genes *groL* and *sigI/σ*^*70*^ were only downregulated by Phe. The transcription of most classical stress tolerance genes was not influenced. Therefore, the regulation of classical HSR and OSR genes was insufficient to explain the cotolerance of *L. rhamnosus* to heat stress and oxidative stress in a low nitrogen source environment.

Interestingly, 71.4% of the enriched overlapping DEGs were related to transport and metabolism of carbohydrate, such as mannose, fructose, ribose, trehalose, galactose, cellobiose and isomaltose. Moreover, most of these genes were similarly expressed in the MRS and NP-MRS groups. Does a high concentration of secondary carbohydrates help *L. rhamnosus* hsryfm 1301 to survive heat stress and oxidative stress? Supplementation of Glc-MRS broth with mannose, galactose, fructose, or ribose did not strengthen the heat stress or oxidative stress tolerance. The use of a secondary carbon source is induced by the carbon source and repressed in carbon catabolite repression (CCR) that the use of secondary carbon sources is reduced in the presence of glucose [5]. CCR is important for competition in natural environments, as selection of the preferred carbon source is a major determining factor in growth rate and therefore competitive success with other microorganisms [25]. In LAB, catabolite control protein A (CcpA) is the global regulatory element in the control of CCR. CcpA also has a key role in the regulation of aerobic metabolism [26, 27]. In the present study, it was also found that genes related to acetate fermentation and formate fermentation were downregulated by both tryptone and Phe, which suggested that nitrogen sources influence CCR in *L. rhamnosus*.

CcpA has been shown to be involved in stress response mechanisms by exerting positive control on the *dnaK* and *groESL* operons in *L. plantarum* [28]. For exponential phase cells, inactivation of *ccpA* impaired both heat stress and cold/starvation stress, but increased oxidative stress tolerance in *L. plantarum* [5]. Replacing glucose with mannose, fructose or ribose not only strengthened the heat stress tolerance, but also improved the oxidative stress tolerance of *L. rhamnosus* hsryfm 1301, which indicated that relieving CCR could help *L. rhamnosus* hsryfm 1301 resist heat stress and oxidative stress. Unlike *L. plantarum*, the CCR in *L. rhamnosus* hsryfm 1301 was conducive to the tolerance of both heat stress and oxidative stress, which could explain the characteristic cotolerance of *L. rhamnosus* to heat stress and oxidative stress. To our knowledge, catabolite responsive elements (*cre*) and fructose-1,6-biphosphate are necessary for achieving CCR [25, 29], and nitrogen sources have not been reported to influence the function of CcpA. The finding that a low nitrogen source environment relieved CCR and then strengthened stress tolerance was interesting and worthy of further study.

## Conclusion

The resistomes of *L. rhamnosus* hsryfm 1301 in the MRS, NP-MRS and Phe-MRS groups were identified. Most of the enriched overlapping DEGs were involved in secondary carbon source transport and metabolism. Replacing glucose by a secondary carbon source rather than adding a secondary carbon source helped *L. rhamnosus* hsryfm 1301 survive heat stress and oxidative stress, suggesting that a low nitrogen source environment strengthened the H-OST of *L. rhamnosus* hsryfm 1301 by relieving CCR. It is a new point of penetration to investigate the relationship between stress tolerance and carbon metabolism. The survival rate of *L. rhamnosus* during spray drying will hopefully be improved by relieving CCR.

## Materials and methods

### Bacterial strain and growth conditions

*L. rhamnosus* hsryfm 1301 was isolated from the gut of a centenarian from Bama, Guangxi Province, China, in 2013 and preserved in the China General Microbiological Culture Collection Center (CGMCC No. 8545) [10]. The strain was cultured in MRS broth (2% (v/v) inoculation) at 37 °C under static incubation.

MRS, NP-MRS (tryptone-free MRS) and Phe-MRS (addition of Phe to NP-MRS with a final concentration of 0.8 g L^−1^) broths were prepared according to our previous study [16]. Sugar supplement media were prepared with the addition of mannose, fructose, galactose, or ribose to the glycoprival MRS medium (Table 1 10 g L^−1^) [30].

**Table 1:** Concentrations of carbon sources in the modified MRS media

Media name	Carbon source/concentration
MRS	Glucose/20 g L^−1^
Gly-MRS	N/A
Glc-MRS	Glucose/10 g L^−1^
Man-MRS	Mannose/10 g L^−1^
Fru-MRS	Fructose/10 g L^−1^
Gal-MRS	Galactose/10 g L^−1^
Rib-MRS	Ribose/10 g L^−1^
Glc + Man-MRS	Glucose/10 g L^−1^, Mannose/10 g L^−1^
Glc + Fru-MRS	Glucose/10 g L^−1^, Fructose/10 g L^−1^
Glc + Gal-MRS	Glucose/10 g L^−1^, Galactose/10 g L^−1^
Glc + Rib-MRS	Glucose/10 g L^−1^, Ribose/10 g L^−1^

### RNA isolation and sequencing

After overnight incubation, *L. rhamnosus* hsryfm 1301 was diluted 50-fold in 50 mL of fresh MRS broth, NP-MRS broth and Phe-MRS broth, and incubated at 37 °C until the optical density at 600 nm (OD_600_) reached 1.8–2.0. Each broth group included 3 replicates. The cells of *L. rhamnosus* hsryfm 1301 were washed with PBS buffer and harvested by centrifugation at 6000×*g* for 10 min at 4 °C. The resulting pellets were immediately frozen in liquid nitrogen.

For RNA extraction, total RNA was isolated using TRIzol Reagent (Invitrogen Life Technologies, Carlsbad, CA, USA). Quality and integrity were determined using a Nano Drop spectrophotometer (Thermo Scientific, Wilmington, DE, USA) and a Bioanalyzer 2100 system (Agilent, Palo Alto, CA, USA). Library construction and RNA-Seq were performed by Shanghai Majorbio Bio-pharm Technology Co., Ltd. (Shanghai, China) as reported in a previous study [31].

The data generated from the Illumina platform were used for bioinformatics analysis. Analyses such as correlation of expression patterns were performed using the free online Majorbio Cloud Platform (www.majorbio.com) from Shanghai Majorbio Bio-pharm Technology Co., Ltd.

### Mapping reads to the reference genome

The quality information of raw data in FASTQ format was calculated, and then the raw data were filtered using Cutadapt (v1.15) software [32]. Then, high-quality clean data were obtained by removing reads containing adapters, reads containing poly-N and low-quality reads. The remaining clean reads were mapped to the annotated genome of *L. rhamnosus* hsryfm 1301 using Bowtie 2 software [10, 33].

### Differential expression analysis

Gene and isoform abundances from paired-end RNA-Seq data were quantified using RSEM (http://deweylab.github.io/RSEM/). To compare the gene expression levels of different genes and different samples, FPKM (fragments per kilobase of exon per million fragments mapped) was used to normalize the expression [34]. DESeq2 software was used to quantify all transcripts with differential expression [35]. Transcripts with |log_2_FoldChange|> 1 (log_2_FC) and *P value* < 0.05 were considered differentially expressed genes (DEGs).

### KEGG analysis

Kyoto Encyclopedia of Genes and Genomes (KEGG, http://www.kegg.jp/) pathway analysis was performed on target genes of differentially expressed mRNAs. KOBAS 2.0 (http://kobas.cbi.pku.edu.cn) was used to identify statistically significantly enriched pathway using Fisher’s exact test. After multiple testing correction, pathways with a *P value* ≤ 0.05 were considered significantly enriched [36].

### Growth of *L. rhamnosus* hsryfm 1301 in broths with different carbon sources

After overnight incubation, *L. rhamnosus* hsryfm 1301 was diluted 50-fold in 5 ml of each fresh modified MRS broth in which glucose was replaced by different carbon sources and incubated at 37 °C. The OD_600_ was measured at 2-h intervals.

### Heat stress and oxidative stress tolerance detection

**Fig. 9:**
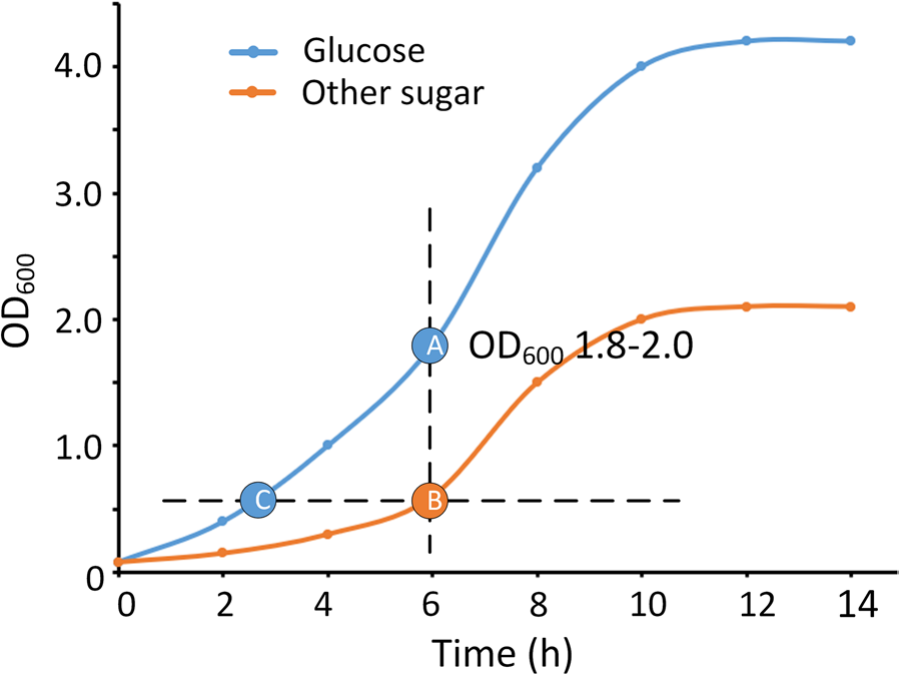
The method applied to define the OD_600_ value at which the stress tolerance was measured. Measurement point A: *L. rhamnosus* hsryfm 1301 cultured in Glc-MRS broth; time, 6 h; OD_600_, 1.8–2.0. Measurement point B: *L. rhamnosus* hsryfm 1301 cultured in modified MRS broth; time, 6 h; OD_600_, measured. Measurement point C: *L. rhamnosus* hsryfm 1301 cultured in Glc-MRS broth; time, at which the OD_600_ was equal to that of measurement point B

After overnight incubation, *L. rhamnosus* hsryfm 1301 was diluted 50-fold in 5 mL of fresh modified MRS broths and incubated at 37 °C. For the modified MRS broth in which *L. rhamnosus* hsryfm 1301 grew as rapidly as in Glc-MRS broth, tolerance detection was implemented when the OD_600_ reached 1.8–2.0 (at 6 h). For the modified MRS broth in which *L. rhamnosus* hsryfm 1301 grew more slowly than in the Glc-MRS broth, tolerance detection was implemented at 6 h (Point B, Fig. 9), and the tolerance in the Glc-MRS broth at the same OD_600_ was detected as a control (Point C, Fig. 9).

The sublethal heat stress and oxidative stress was determined according to the method described previously [15]. (1) **For **oxidative stress. The samples were supplemented with 4.0 mM H_2_O_2_, and then the samples were incubated at 37 °C for 1 h. (2) For heat stress. The samples were incubated at 55 °C in a water bath for 1 h. The colony-forming unit (CFU) counts of the treated samples were calculated according to a previously described drop plate technique [30].

### Statistical analysis

Significant differences between two values were evaluated by unpaired Student’s *t test* (*P* < 0.05).

### Nucleotide sequence accession numbers

The whole genome sequence of *L. rhamnosus* hsryfm 1301 was deposited into the National Center for Biotechnology Information (NCBI) under the accession numbers CP044228 (chromosome) and CP044229 (plasmid pGu1301). The raw sequence data reported in this paper have been deposited in the Genome Sequence Archive in National Genomics Data Center [37, 38], China National Center for Bioinformation / Beijing Institute of Genomics, Chinese Academy of Sciences (accession number: CRA007167, experimental number: CRX455121-CRX455129), which are publicly accessible at https://bigd.big.ac.cn/gsa/browse/CRA007167.

## Data Availability

The data that support the findings of this study are openly available in the Genome Sequence Archive in National Genomics Data Center at https://bigd.big.ac.cn/gsa/browse/CRA007167

## Abbreviations

LAB: Lactic acid bacteria
H-OST: Heat stress and oxidative stress tolerance
OD: Optical density
DEGs: Differentially expressed genes
KEGG: Kyoto Encyclopedia of Genes and Genomes
FPKM: Fragments per kilobase of exon per million fragments mapped
CFU: Colony-forming unit
HSR: Heat shock response
OSR: Oxidative shock response
CCR: Carbon catabolite repression
